# Parametric analysis of SARS-CoV-2 dose-response models in transportation scenarios

**DOI:** 10.1371/journal.pone.0301996

**Published:** 2024-06-12

**Authors:** Yuxuan Wu, Sirish Namilae, Ashok Srinivasan, Anuj Mubayi, Mathew Scotch

**Affiliations:** 1 Embry-Riddle Aeronautical University, Daytona Beach, Florida, United States of America; 2 University of West Florida, Pensacola, Florida, United States of America; 3 QVIA, Durham, North Carolina, United States of America; 4 Arizona State University, Tempe, Arizona, United States of America; Tsinghua University, CHINA

## Abstract

Transportation systems involve high-density crowds of geographically diverse people with variations in susceptibility; therefore, they play a large role in the spread of infectious diseases like SARS-CoV-2. Dose-response models are widely used to model the relationship between the trigger of a disease and the level of exposure in transmission scenarios. In this study, we quantified and bounded viral exposure-related parameters using empirical data from five transportation-related events of SARS-CoV-2 transmission. Dose-response models were then applied to parametrically analyze the infection spread in generic transportation systems, including a single-aisle airplane, bus, and railway coach, and then examined the mitigating efficiency of masks by performing a sensitivity analysis of the related factors. We found that dose level significantly affected the number of secondary infections. In general, we observed that mask usage reduced infection rates at all dose levels and that high-quality masks equivalent to FFP2/N95 masks are effective for all dose levels. In comparison, we found that lower-quality masks exhibit limited mitigation efficiency, especially in the presence of high dosage. The sensitivity analysis indicated that a reduction in the infection distance threshold is a critical factor in mask usage.

## Introduction

There has been significant concern regarding the spread of SARS-CoV-2 on transportation systems. The earliest superspreading events were associated with transportation on cruise ships [1]. Multiple incidents of secondary infection spread have been recorded in various transportation systems including airplanes [2], cruise ships [3], trains [4], and buses [5]. Transportation systems by their very nature involve high-density crowds in relatively small spaces, with limited ventilation. Moreover, geographically diverse groups of people with variations in susceptibility (impacted by vaccination and exposure history) often converge in close proximity. Early preventative measures included reducing or stopping air-travel and other modes of travel to and from affected regions [6]. Economic disruption in reducing travel can be mitigated to some extent by understanding SARS-CoV-2 spread in transportation systems and designing preventative measures for mitigation. Examples include mask usage mandates and guidelines aboard airplanes from the Centers for Disease Control and Prevention (CDC) [7], and social distancing approaches such as keeping middle seat vacant on airplanes [8]. Mathematical modeling can be utilized to develop insights into such approaches. For example, using computational modeling, we recently analyzed the relative effectiveness of masks and social distancing in air travel [9–11], and effective boarding practices [12, 13].

SARS-CoV-2 is primarily transmitted by direct contact with pathogen droplets or aerosols released by a proximate infective person, which correlates with the exposure time and distance between susceptible and infective individuals [14]. Congregation of many people, such as in transportation systems, combined with a high viral load in the pathogen dose, can result in a high number of secondary infections [15].

While modeling combined with empirical observations is effective in analyzing infection-spreading events, there are limitations with respect to data quality (including availability) and the modeling approaches that use the data. Variations in human behavior with respect to mask usage, social distancing, and personal hygiene lead to a wide variety of situations. This, combined with biological stochasticity, results in a vast parameter space. We recently developed algorithms for parameter sweeps that can effectively cover the parameter space with fewer simulations [16, 17]. Empirical data can also help to reduce this parameter space. While there are numerous anecdotal instances and news articles on infection spread during transportation, the paucity of contact tracing has limited the data available on infection spread events associated with transportation systems. In this study, we utilized relatively limited empirical data to estimate and bound the parameters related to pathogen dose and its response to proximate susceptibility.

Among the diseases mainly driven by proximity, SARS-CoV-2 can be transmitted over relatively long distances, as high as 12 ft [18]. Therefore, it is necessary for mathematical models to address variations in the viral load with distance for high-resolution phenomenological modeling. In the context of transportation systems, this means that the models should account for the fact that a susceptible passenger seated 3-ft away is more likely to be infected than one seated 10 ft away. The number of possible infections can be modeled based on the detailed locations and trajectories of individuals and the probability of transmission. In such scenarios, one of the main factors determining whether the infection can be successfully initiated is the amount of pathogen (dose) transmitted from the infected to susceptible individuals [19]. Transmission depends on the characteristics of pathogens, such as droplet or aerosol dimensions and viral load [20], environmental factors such as ventilation, mask usage, and distance between individuals [21], and the duration of exposure [22]. Classical dose-response models are widely used to study such problems by modeling the relationship between the successful trigger of diseases and the amount of transmitted dose. Several dose-response models [23–25] estimate the probability of infection for susceptible individuals that are spatially located in the proximity of infective individuals. Given the novelty of SARS-CoV-2 and unique considerations in transportation systems, these models and empirical data need to be analyzed to find an adequate model and corresponding parameter ranges.

Researchers use infection models in general and dose-response relationships for different types of modeling purposes: (i) descriptive or causal models that explain the mechanisms of a past infection outbreak, (ii) predictive modeling of potential outbreaks, and (iii) prescriptive models that guide interventions. For descriptive or causal models, one would fit the model to the infection outbreak data and examine if the best parameter choice explains the result adequately. For predictive models, one would use a validated model and model parameters for predicting an outbreak in a new situation. For prescriptive purposes, one would generate a variety of possible scenarios and identify interventions that are effective under all these scenarios, so that the intervention is robust against the inherent uncertainties due to biological stochasticity and human behavior. This would require identification of a set of models and parameters that yield different outcomes in the absence of the intervention so that we have a rich set of possible scenarios to evaluate.

The main contribution of our study is toward epidemiological mitigation policy designs. The continuing reemergence of various strains of SARS-CoV-2 and other respiratory infectious diseases in the post-pandemic period and the related attention towards intervention strategies further necessitates this study. To determine effective interventions, the simulation of worst-case or bad-case scenarios is required and the intervention performance needs to be investigated. This can be challenging for new epidemics, as the mechanisms of the outbreak and the specific model to describe the outbreak is not clear. Furthermore, model parameters representing a large outbreak are not known either. Therefore, to determine effective interventions, many different models, representing different possible mechanisms, with variation in parameters, need to be studied and calibrated against significant outbreak to identify different scenarios that ought to be simulated. With this motivation, we: (a) Quantify the viral dose-related parameters in multiple transportation scenarios by analyzing empirical data from related infection spread events. (b) Compare the various dose-response models and assess their applicability in transportation scenarios. (c) Apply the validated models to parametrically analyze the spread of infection and the effect of mask usage in generic transportation modes, including airplanes, railway coaches, and buses.

## Methodology

### Dose response models

Consider a situation with M infectious individuals in a total population of N individuals with the potential for close proximity contact. When an infective individual *i*_*M*_ and susceptible individual *j*_*N*_ are considered to be in contact, the amount of dose d transmitted during the contact duration (Δ*t*) can be computed as:

$$d{\left(\Delta t\right)}_{j_{N},i_{M}}=\beta \cdot \Delta t\cdot \sum_{i_{0}}^{M}{\left(1-\frac{r_{ji}}{r_{thr}}\right)}^{\alpha }$$ (1)

where β defines the infectivity of the pathogen (probability of successful pathogen transmission per contact), and *r*_*ij*_ is the actual distance between two individuals. *r*_*thr*_ is the threshold distance beyond which infection is not transmitted. *α* is the deterioration rate of the pathogen within the spreading radius, and *β* defines the infectivity of the pathogen.

Susceptible individuals with similar dosage exposure levels respond differently because of the biological stochasticity and variations in the vulnerability and receptivity of individuals. The probability of infection for a given exposure to dose (d) was modeled using dose-response models. There have been many applications of dose-response models for the quantitative evaluation of disease transmission from sources, such as sewage, polluted animals, and pathogen-containing aerosols [26–28]. Previous studies have compared the effectiveness of these models, such as the dose-response accuracy [29] and modeling simplicity [30] for various diseases using known biomedical da ta. However, the application of these models to SARS-CoV-2 has been limited because of the novelty of the virus. Watanabe et al. [30] developed the parameters for an exponential dose-response model for the SARS-CoV outbreak in the early 2000’s using datasets of infected mice to analyze the outbreak in a Hong Kong apartment complex. Zhang and Wang [31] evaluated the effect of aerosol transmission viral load on the infection risk of SARS-CoV-2 using an exponential model. Parhizkar et al. [32] developed an infection risk assessment platform with an exponential model in which the estimation suggested a reasonable match with available metadata from outbreaks.

Here, we formulate an infection risk model based on previously established dose-response relations and parameterize it using infection spread scenarios in the context of transportation. Among all available models, the exponential and beta-Poisson models are two commonly used mathematical models, whereas the Weibull model is an effective empirical model [33]. Other less-frequently used models, such as Log-logistic model [34], and Log-Probit model [34], both of which are mainly driven by empirical data will not be compared in this study due to the concern of epistemic uncertainties arising from model parameterization. The mathematical abstractions of three considered models are listed in Table 1.

**Table 1:** Mathematical formulations used for dose response models.

Model	Formulation	Parameter interpretation	Reference
Exponential	*P*(*d*) = 1 − *exp*(−*rd*)	r: probability for one pathogen cell to trigger a response	[35]
Beta-Poisson	$\text{P}\left(d\right)=1-{\left(1+\frac{d}{b_{2}}\right)}^{-b_{1}}$	*b*_1_:pathogen infectivity; *b*_2_: statistical parameter (shape)	[36, 37]
Weibull	$P\left(d\right)=1-exp\left(-q_{1}d^{q_{2}}\right)$	*q*_1_: pathogen infectivity; *q*_2_: statistical parameter(shape)	[38, 39]

In the exponential model, the pathogen infectivity parameter r is often assigned a fixed value to represent the probability of successful transmission triggered by a single-shot dose [35]. The beta-Poisson model is usually considered an extension of exponential model, where the pathogen infectivity follows a Γ-distribution instead of a fixed value [35]. For simplicity, the beta-Poisson model is generally approximated in the format shown in Table 1, with numerical requirements of *b*_2_ ≫ 1 and *b*_2_ ≫ *b*_1_ [40]. Further, the values of b1 and b2 can be bounded as 0.05 < *b*_1_ < 2 and *b*_2_ > (22*b*_1_)^0.5^ to cover many modeling studies [41]. The Weibull model defines infectivity using a Weibull distribution and has been widely used as an empirical model to fit the available data for diseases such as influenza (A/H1N1 2009) [42] and respiratory cancer [43].

In this study, we parameterized the exponential, beta-Poisson, and Weibull models using the transmission events described section 3.2 Parameterization Contexts. The models were then used to study the spread of infection in generic transportation scenarios with varying doses and mask usage levels.

### Parameterization contexts

To parameterize the dose-response models to specific events of SARS-CoV-2 spread, details of the location of the infected individuals and the duration of exposure are needed. While there are numerous studies that provide overall information on the number of infections, relatively few studies include detailed location maps. These events were used to parameterize the dose-response parameters in Eq (1) and Table 1. Four of these events occurred in the transportation context. Additionally, we included one well-studied infection event in a restaurant providing references for parameterizing the other events.

**Fig 1:**
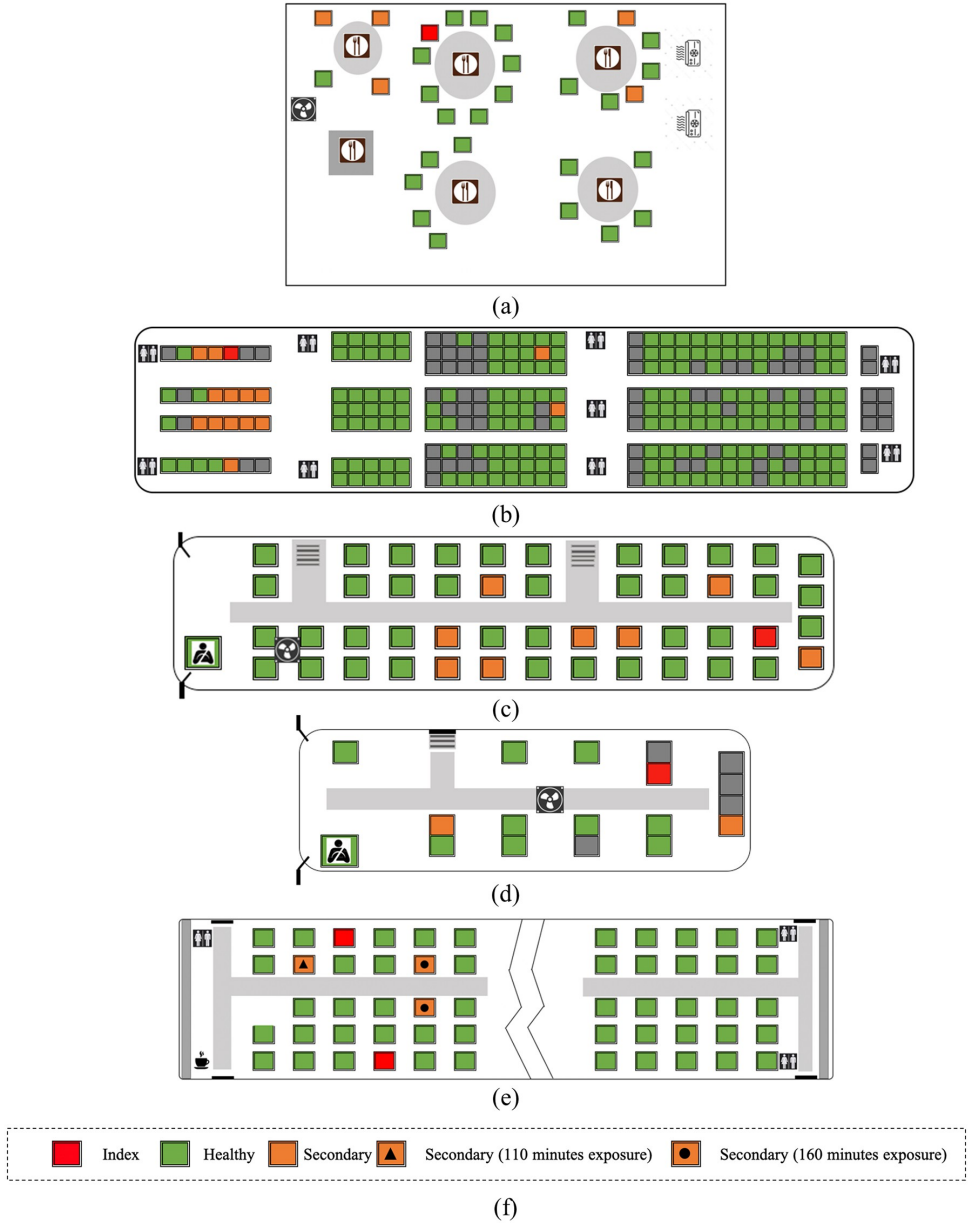
Schematic infection maps of (a) Guangzhou restaurant (b) UK flight (c) Wuhan large bus (d) Wuhan mini-bus, and (e) Harbin train used for parameterization (f) legend label [44, 45, 48, 49].

A gathering at a restaurant in Guangzhou, China was identified as a superspreading event, where one known infective customer resulted in 4 to 5 secondary infections with an exposure duration of one hour [9, 44]. The event occurred in the early stages of the pandemic; therefore, there was no mask usage. The data for the position of everyone in the restaurant, duration of exposure, and infection status before and after the event are documented in Lu et al. [44]. The second event we considered was a 10-hour flight from London to Hanoi on March 1, 2020, with 201 passengers onboard [45]. One infectious individual seated in the first class resulted in 14 secondary infections in the aircraft, 12 were seated in close proximity to the index case in the first class section and two were in the economy cabin. Again, there was no mask usage on this flight. The London-Hanoi flight contains relatively high infective dose involving with 92% nearby passenger infection rate [46], and 7.4% boarding infection rate associated with geo-prevalence and mask wearing policies [47]. While there are other flights involving transmissions, we specifically use London-Hanoi flight to assess the high-dose transmission scenarios. Similarly, we select the following events to assess the intermediate and low dose levels. The third and fourth events used for quantifying the intermediate dose involve the same index case: traveling first on a larger bus and then on a mini-bus near Wuhan in China [48]. The larger bus contained 48 passengers onboard and 9 secondary infections were found after a 2.5-hour travel period, among which 8 infections were identified directly related to the trip whereas one infection was suspected to be infected elsewhere. This was followed by a one-hour trip in a mini-bus with 12 passengers, which resulted in two secondary infections. There was no mask usage in any of these cases. Although the Wuhan large bus and the mini-bus event have the same index case, the layout of susceptible individuals is different, therefore they are separate events. These two events provided an infectivity range for the same index cases. The fifth event for low-dose analysis is the multistage transmission of infection on a train starting from Harbin, China [49]. The first stage of infection involves 110 minutes of exposure, which results in one secondary infection. The second stage involves two secondary infections with 160 minutes of exposure from the same two index cases. There was no temporal overlap between the two infection events. The 110-minute portion of the train journey is use for parameterization as it falls due to the high possibility of close-proximity induced infections maximally excluding the effect of possible passenger movements due to the longer duration. Fig 1 schematically shows the location of the index case and secondary infections for these five events.

Parameters involved in all three models are categorized into two folds, dose transmission parameters that mathematically describe the transmission mechanism including α, β, and *r*_*thr*_, and dose survivability parameters that statistically shape the survivability distribution of pathogen during triggered transmissions including *b*_1_, *b*_2_ parameters in the beta-Poisson model and the *q*_1_, *q*_2_ parameters in the Weibull model. The dose survivability parameters for the exponential model are implicitly accounted during the quantification of dose in each parameterization context.

Given the seating arrangement and the resulting distance between the reported index case and susceptible individuals. Contact is considered to occur when the distance between two individuals (*r*_*ij*_) is less than *r*_*thr*_. It has been reported that suspended SARS-CoV-2 droplets can be collected up to 4 meters away from an index person. The value *r*_*thr*_ is bounded based on physical considerations of the aerosol and particle spread mechanisms [50]. We vary the values of α, β, and *r*_*thr*_ using a standard grid search minimization algorithm to compute the infection probability of each susceptible individual. The sum of the infection probabilities provides the total number of infections for a given event. The model parameter set that produces the correct number of total infections within tolerance and predicts high probabilities for actual secondary infections are chosen to be the model parameters as described by Eq 2.


$$\overset{⌢}{\Theta }=min_{\Theta }\left(\;,N_{observed}-N_{\text{mod}el}\right)$$ (2)

The above method was directly used for the exponential model. For the beta-Poisson and Weibull models, additional dose survivability parameters (*b*_1_, *b*_2_ for the beta-Poisson model and *q*_1_, *q*_2_ for the Weibull model) are required to define the models. We observed that transmission parameters have a greater impact on the spread of infection than survivability parameters. Therefore, transmission parameters were prioritized, using the parameter set from the exponential models as an initial guess to establish an acceptable range. Survivability parameters from existing literature [51] were used as an initial approximation in this process. This parameter set was considered as a basis to further fine-tune all parameters including survivability parameters using the same minimization approach, to obtain the final parameter set.

The parameter values corresponding to parameterization that satisfies the total number of infections and gives a high probability of infection for the actual cases within the fitting error criteria (less than ± 1 infected) for the five different events are summarized in Table 2. The use of numerous transmission events provides the variation in the range of dose. The raw data used for parameterization including seating arrangements and exposure durations along with the best fitting parameters and the fitting errors are provided in the S1 Data.

**Table 2:** Model fitting parameters from available superspreading events.

Model Parameterization	Guangzhou Restaurant [44]	UK flight [45]	Wuhan Large bus [48]	Wuhan Mini bus [48]	Harbin Train [49]
Exponential	*α* = 2.5	*α* = 2.4	*α* = 3.3	*α* = 3.8	*α* = 6.3
	*β* = 0.15	*β* = 0.2	*β* = 0.02	*β* = 0.03	*β* = 0.007
	*r*_*thr*_ = 3.5m	*r*_*thr*_ = 3.5m	*r*_*thr*_ = 3.5 m	*r*_*thr*_ = 3.5m	*r*_*thr*_ = 2m
Beta-Poisson	*α* = 1.6	*α* = 1.6	*α* = 3.6	*α* = 2.9	*α* = 2.5
	*β* = 1.45	*β* = 1.4	*β* = 0.6	*β* = 0.5	*β* = 0.06
	*r*_*thr*_ = 3.5m	*r*_*thr*_ = 3.5m	*r*_*thr*_ = 3.5m	*r*_*thr*_ = 3.5m	*r*_*thr*_ = 2m
	*b*_1_ = 2.3	*b*_1_ = 2.3	*b*_1_ = 2.3	*b*_1_ = 2.3	*b*_1_ = 2.3
	*b*_2_ = 52.3	*b*_2_ = 52.3	*b*_2_ = 52.3	*b*_2_ = 52.3	*b*_2_ = 52.3
Weibull	*α* = 0.53	*α* = 1.2	*α* = 2.9	*α* = 2	*α* = 2.2
	*β* = 1.5	*β* = 1.45	*β* = 1.5	*β* = 1.45	*β* = 0.3
	*r*_*thr*_ = 3.5m	*r*_*thr*_ = 3.5m	*r*_*thr*_ = 3.5m	*r*_*thr*_ = 3.5m	*r*_*thr*_ = 2m
	*q*_1_ = 0.0016	*q*_1_ = 0.0016	*q*_1_ = 0.0016	*q*_1_ = 0.0016	*q*_1_ = 0.0016
	*q*_2_ = 1.487	*q*_2_ = 1.487	*q*_2_ = 1.487	*q*_2_ = 1.487	*q*_2_ = 1.487

### Application to transportation scenarios

**Fig 2:**
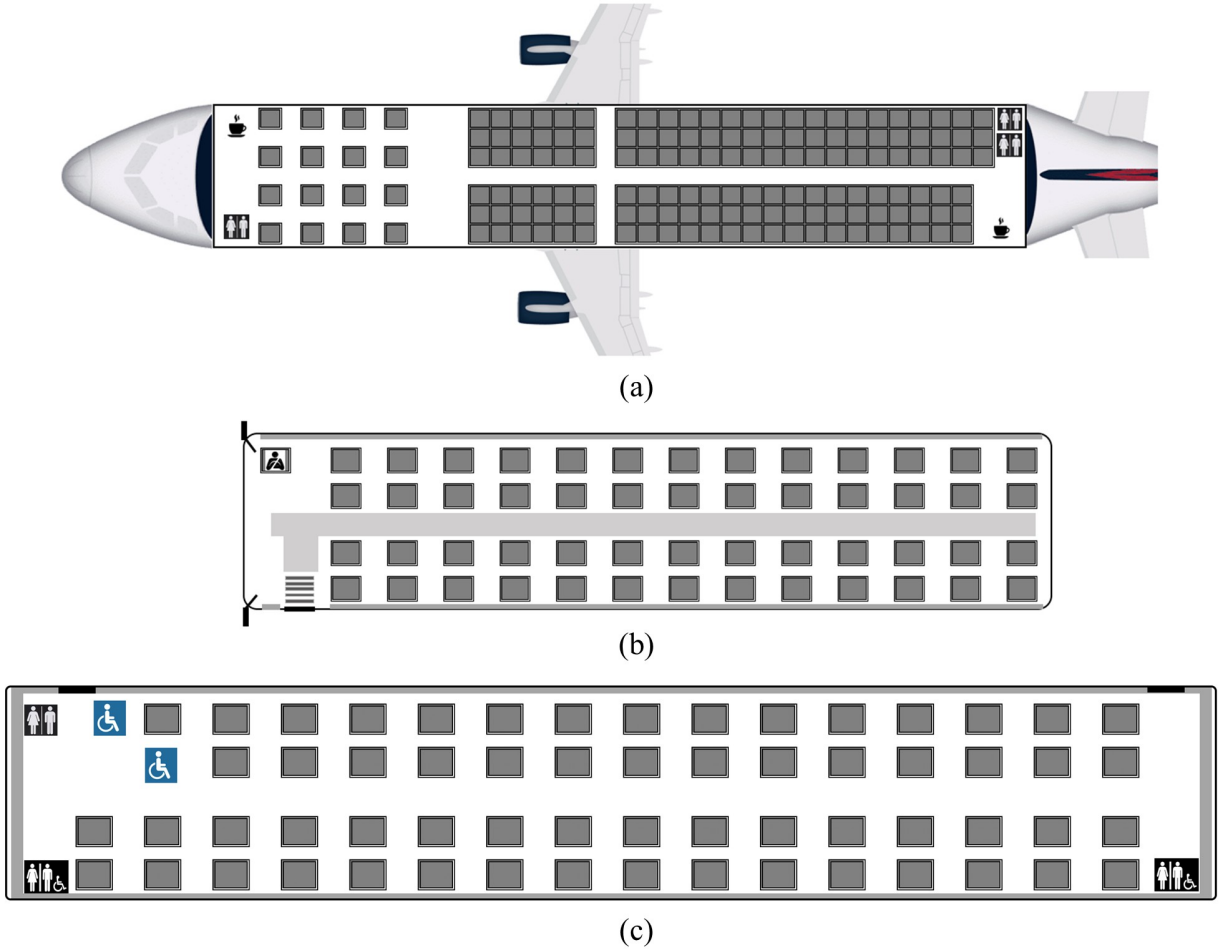
Schematic of modeling scenario of a generic (a) single aisle aircraft, (b) bus (c) railway coach.

We applied the dose-response models to three generic transportation modes (Fig 2) including a:

A two-aisle airplane modeled with a layout similar to Airbus A320 aircraft, with 157 passengers including 16 in the business cabin and 141 in the economy cabin. The total duration of the flight was simulated to be two hours, which corresponds to common regional flights.

A bus with a similar layout as Greyhound bus, with 52 passengers and one driver. We used a duration of two hours to be consistent with airplane case.

A railway coach with a seating layout based on Amtrak passenger coach with 61 passengers uniformly seated facing the direction of movement of the train. A two-hour duration is used here as well.

In our earlier work, we incorporated the effect of pedestrian movement on the spread of infectious diseases [9–13]. We found that pedestrian movement such as the boarding and deplaning of an aircraft can explain some empirical observations regarding the location of infected passengers [9]. However, the primary contribution to secondary infections was the colocation of passengers in proximity over an extended period. In our five transportation scenarios studied in this current work, we focused on dose variation; therefore, we do not consider the effect of pedestrian movement. Our model methodology can be easily extended to incorporate human movement in future studies.

We also model the impact of mask usage. Note that all the parameters obtained by model fitting are for transmission events with no mask usage. With the application of masks, pathogens can be filtered so that the amount of pathogen dose and range of spread are lower depending on the mask quality. We reduced the parameters β and *r*_*thr*_ in Eq (1) by a factor corresponding to the mask quality to simulate mask usage. For example, studies have indicated that high-quality N95 masks with a normal fit are approximately 97% effective in preventing leakage [52]. Cloth masks, such as cotton bandana “folded surgeon’s general style, have a filtration efficiency of up to 50%. In addition, reports indicate that the distance traveled by respiratory droplets is halved with the use of surgical masks [53]. Based on these studies, we performed parameter sweeps to investigate the influence of mask usage on the spread of infection.

## Results

### Parameterization of dose response models

The parameter values for the five different events for the three models are summarized in Table 2. Although the Wuhan large bus and the mini-bus event have the same index case, the layout of susceptible individuals is different, therefore they are separate events. For the Harbin train, one infection transmission occurred during a 110-minute part of the journey, with two more infections occurring in a separate 160-minute portion of the train ride, with the same index cases. The parameterization in Table 2 is based on the 110-minute portion of the train journey. As mentioned earlier, the *b*_1_, *b*_2_ parameters in the beta-Poisson model, and the *q*_1_, *q*_2_ parameters in the Weibull model were obtained using the similar approach and were close to parameters reported in a previous study on SARS-CoV-2 transmission [51]. These parameters describe the shape of the probability distribution for a given dose. The values shown in Table 2 correspond to parameterization that satisfies the total number of infections and give a high probability of infection for the actual cases within the fitting error criteria. Furthermore, the variation of *r*_*thr*_ is bounded based on aerosol and droplet mechanisms [50]. The use of numerous transmission events provides the variation in the range of dose.

The corresponding parameters of each model were further compared based on the amount of dose d generated in each scenario as formulated in Eq 1. There are variations in these parameterizing scenarios, i.e., the event duration, the number of infective individuals. In addition, biological stochasticity and human behavioral factors lead to dose variations. Furthermore, the magnitude of the dose required to replicate the observed empirical data is different for the three models because of the differences in the distribution scales which are dependent on the model parameters. To compare the dose levels between the models and scenarios, we normalized the unit dose (per unit time, per index case) to the UK flight scenario as shown in Table 3. We observed that the Guangzhou restaurant and the UK flight scenario contain the highest level of dose, followed by the Wuhan large bus and mini-bus scenarios, and the Harbin train scenario was the lowest. The ranking of the scenarios is consistent across the three models, with minor variations. Among the three models, Weibull model deviated the most from the other two models.

**Table 3:** Comparison of the normalized dose d in each parameterization scenario.

Parameterization		Guangzhou Restaurant [44]	UK flight [45]	Wuhan Large bus [48]	Wuhan Mini bus [48]	Harbin Train [49]
Number of infective		1	1	1		2
Exposure/min		20~73 (average = 44)	600	150	60	160
Dose per min per index	Exponential	0.483	1.000	0.198	0.114	0.019
	Beta	0.855	1.000	0.525	0.232	0.049
	Weibull	2.007	1.000	1.215	0.692	0.199

Based on the above analysis we refer to the dose levels that are related to the Guangzhou restaurant and the UK flight as high dose parameters, the Wuhan large bus and mini-bus as intermediate dose parameters, and the Harbin train as low dose parameters. Next, we proceed to use the parameterization in each of the above contexts to analyze the other scenarios. This provides a hypothetical infection analysis of these scenarios utilizing the dose range ranked previously. In Table 4, the first three rows correspond to modeling the Guangzhou restaurant scenario using parameterizations corresponding to the five scenarios using the three models. Similarly, the next three rows correspond to modeling the UK flight with different parameterizations. The subsequent rows correspond to the other three scenarios. Here, the UK flight and Guangzhou restaurant both correspond to high dose parameters, therefore when the parameters corresponding to one of those scenarios is used to model the other, there is close agreement in the number of infections to the observed data. When either of these parameterizations is used to model the Wuhan bus or Harbin train scenarios, the number of infections is significantly higher. For example, if the high dose parameters corresponding to the UK flight scenario are applied to the Wuhan bus, the models predict that there would be 16–18 potential infections. Empirical data for this intermediate dose scenario shows eight infections. If the dose in this situation were like that of Guangzhou restaurant, the model calculates that there would be 18 infections, and if the dose was lower, corresponding to that of Harbin train, there would be about one new infection. The diagonal of the table corresponds to the results of parameterization of the concerned scenario; therefore, it closely matches the empirical results. This analysis shows that the variation in dose significantly affects the estimation of infections.

**Table 4:** Cross-validation of parameters in scenarios.

Scenarios and Models		Guangzhou Restaurant [44]	UK flight [45]	Wuhan Large bus [48]	Wuhan Mini bus [48]	Harbin Train [49]
Guangzhou Restaurant (4*)	Exponential	4.3	5.1	0.7	0.7	1.7
	Beta	4.0	4.0	0.7	0.9	0.04
	Weibull	4.0	1.8	0.5	0.9	0.03
UK flight (12*)	Exponential	11.3	11.9	5.1	5.0	1.0
	Beta	12.0	12.0	4.8	5.7	1.1
	Weibull	9.0	11.8	5.5	8.2	1.4
Wuhan Large Bus (8*)	Exponential	17.5	18.3	8.0	8.9	0.8
	Beta	16.7	16.6	8.0	8.6	1.3
	Weibull	18.1	14.2	8.1	10.7	1.3
Wuhan Mini Bus (2*)	Exponential	6.7	7.4	1.7	2.0	0.3
	Beta	5.8	5.7	1.8	2.0	0.2
	Weibull	4.8	3.1	1.5	2.0	0.2
Harbin Train (2*)	Exponential	44.3	48.1	16.9	18.1	2.0
	Beta	28.8	28.6	12.9	14.1	2.0
	Weibull	12.1	22.7	29.9	27.8	2.1

*Actual number of infections

Based on the results in Tables 3 and 4, we can conclude that the three models based on exponential, beta-Poisson and Weibull distributions adequately reproduce the empirical data with appropriate parameterization. When parameterization based on one scenario is applied to other cases (Table 4), one can observe that exponential and beta-Poison models produce similar results while Weibull model indicates fewer infections especially at higher numbers. This is because the tail of the probability density distribution of Weibull model using the specific scalar and shape parameters is different from that of the exponential and beta-Poisson distributions. The cross validation presented in Table 4 suggests that Weibull model can reproduce the parameterizing scenarios but produces outcomes different from exponential and beta-Poisson models in other scenarios. This is critical in the context of prescriptive modeling, where we wish to examine if the different models produce a different set of outputs so that a variety of scenarios would be generated. Next, we apply the models to generic transportation modes and study the impact of mask usage. In subsequent sections, we present the results using the exponential and Weibull models.

### Model application to generic transportation contexts

**Fig 3:**
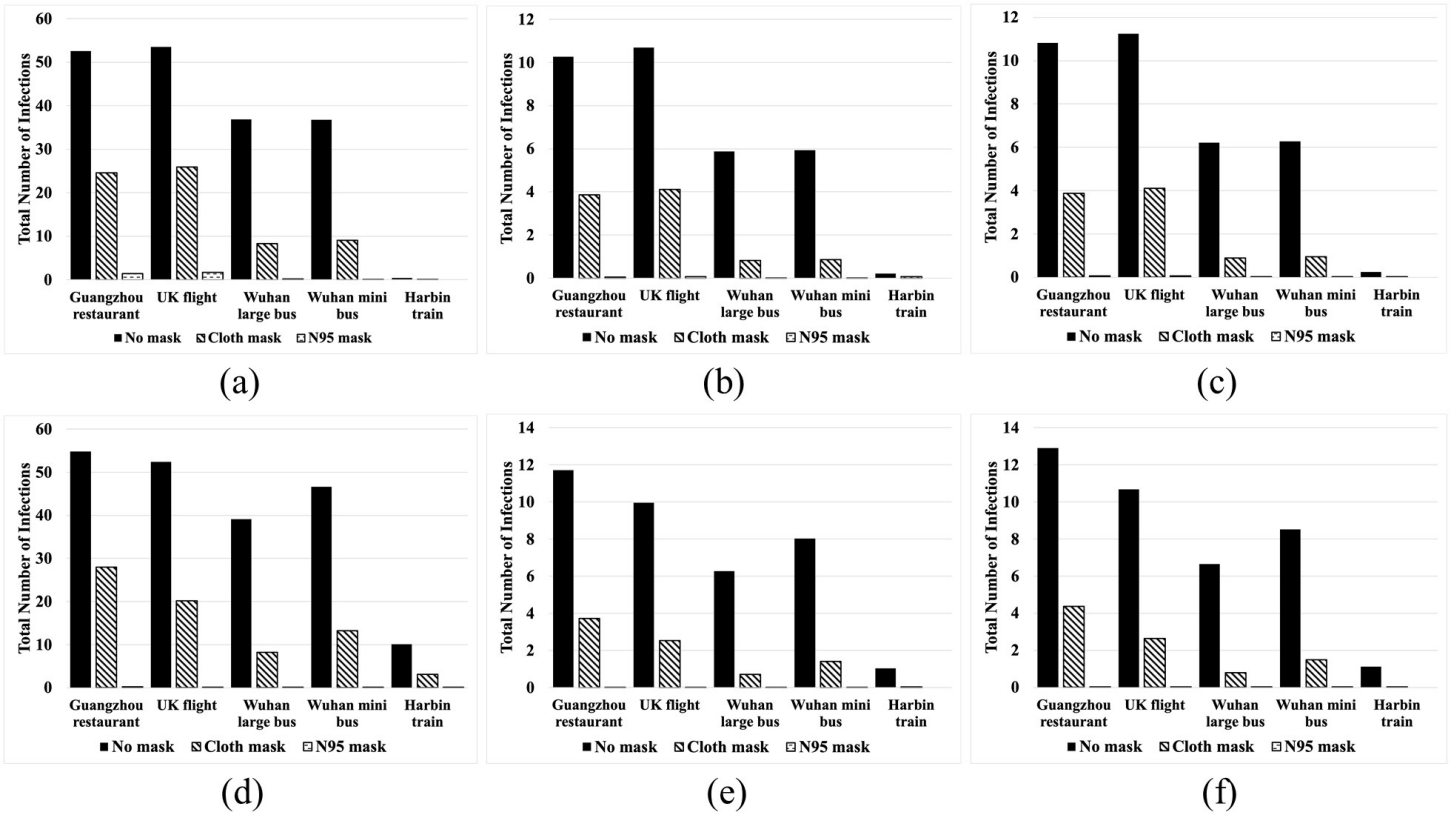
Total infection in (a) single aisle aircraft (b) bus (c) railway coach using the exponential models and in (d) single aisle aircraft (e) bus (f) railway coach using the Weibull models.

The dose level of the infected individual varied from high to low based on the parameterizations described in section 4.1. For generic situations the position of the infective individual is not known apriori, therefore, we vary the location of the infective individual through all possible positions and found the average number of infections with each dose level (Fig 3). Also shown is the effect of all passengers using a cloth mask and an N-95 mask, compared to a no-mask situation. For all three situations, the highest and fewest numbers of infections are observed from the highest level of dose corresponding to the Guangzhou restaurant and the UK flight parameters, and the lowest level of dose corresponding to the train parameters, respectively. The UK flight corresponded to a two-aisle configuration, and the infective was located in the more spacious First-class cabin. The dose response model suggested that an infective with similar dose level in a more densely packed economy cabin of a single aisle aircraft would generate higher number of infections. If the infective was modeled with low dose train parameters, the number of infections is much lower. Also, mask usage, especially N-95 mask usage significantly reduces the number of infections even with a high dose infective.

Similar trends can be observed for the bus and railway coach as shown in Fig 3(b) and 3(c). The number of infections in the railway coach varied from 11 to 1 by varying the dose levels. Using cloth masks reduced the infections to 4 and using N95 masks reduced the infections to zero for the high dose infective.

### Parametric variation

**Fig 4:**
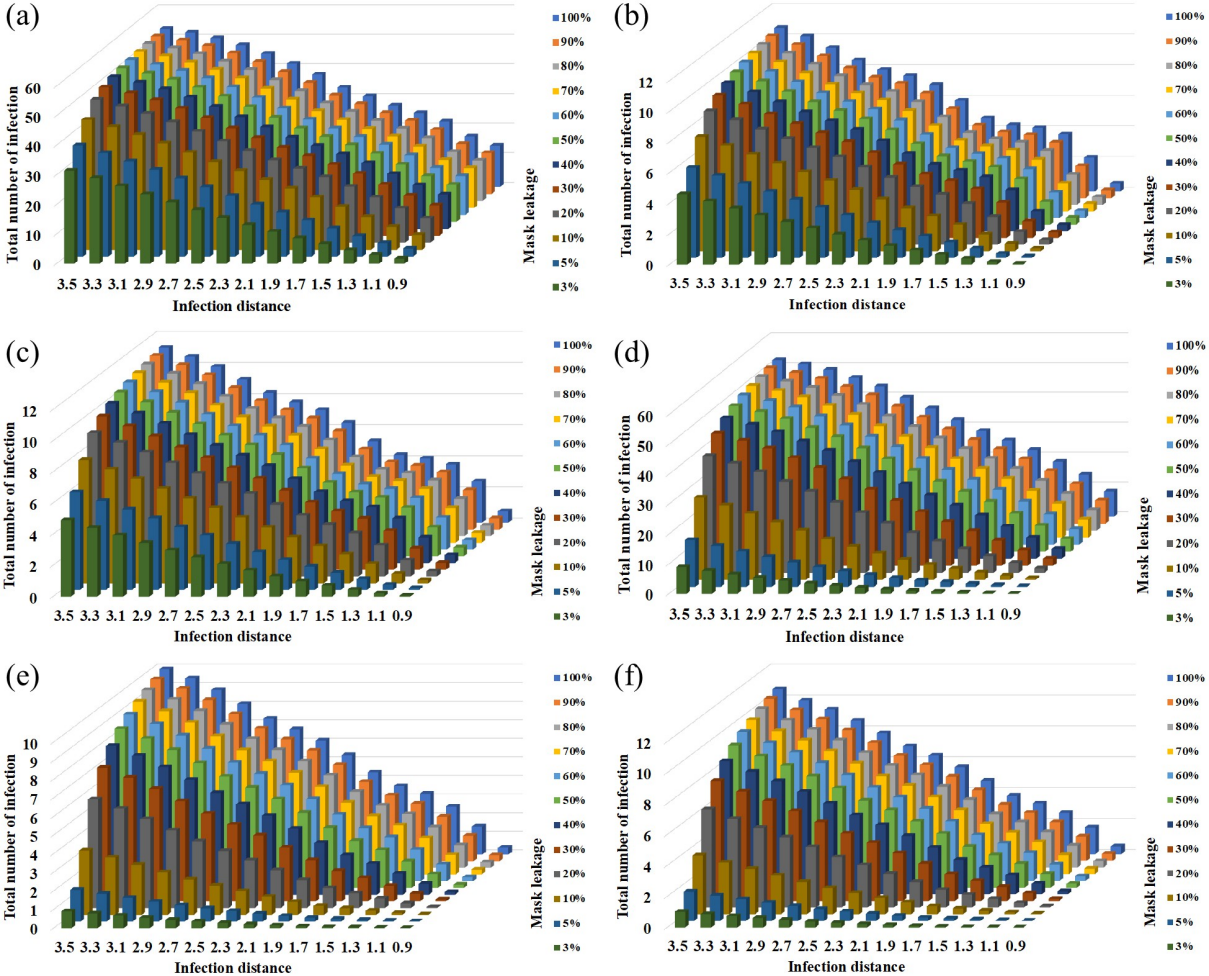
Parameter sweep with high dose—UK flight parameters using the exponential models for (a) single aisle airplane (b) bus (c) railway coach and using the Weibull models for (d) single aisle airplane (e) bus (f) railway coach.

The efficiency of masks varied depending on the mask type, manufacturing quality and facial-fitting. These factors affect the filtration efficiency as well as the distance threshold. A parameter sweep by varying the mask efficiency and distance threshold from a no-mask case to a well-fitting N-95 masks provides a clearer understanding of the role of dose and mask usage in infection spread. Fig 4 shows the parameter variation of mask efficiency and threshold distance for the three cases considered here using high dose (UK flight) parameters. All three cases show a consistent reduction in infections with mask usage, with well-fitting N-95 masks resulting in close to zero infections. Lower quality masks exhibit a limited mitigation efficiency especially for high dose conditions. The reduction in number of infections varies more gradually with improving mask quality in high dose situations (Fig 4) compared to low dose situations.

**Fig 5:**
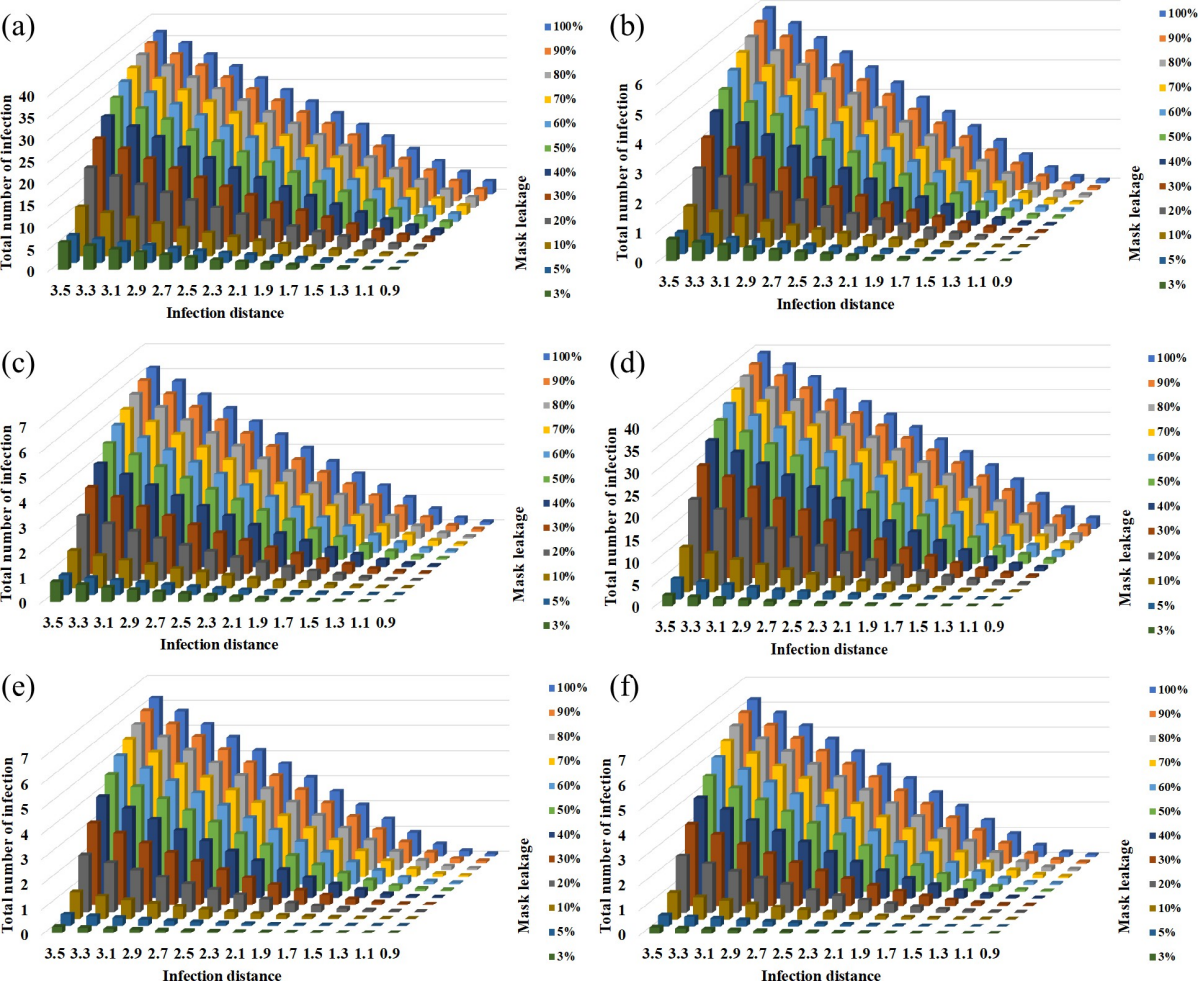
Parameter sweep with intermediate dose—Wuhan large bus parameters using the exponential models for (a) single aisle airplane (b) bus (c) railway coach and using the Weibull models for (d) single aisle airplane (e) bus (f) railway coach.

Fig 5 shows the parameter variation with a low dose infective utilizing the Wuhan large bus parameters for the three applications. In contrast with the “convex” shape from the previous high dose application, using the low dose parameters exhibits a concave shape (see Fig 5a). This suggests that lower quality masks can also provide adequate mitigation for low dose situations. Using higher quality masks reduces the number of infections to zero for a significant part of the parameter space as shown in Fig 5(a). The results follow a similar trend for beta-Poisson and Weibull dose response models, with Weibull exhibiting difference from the other two models in high infection cases.

### Sensitivity analysis

**Fig 6:**
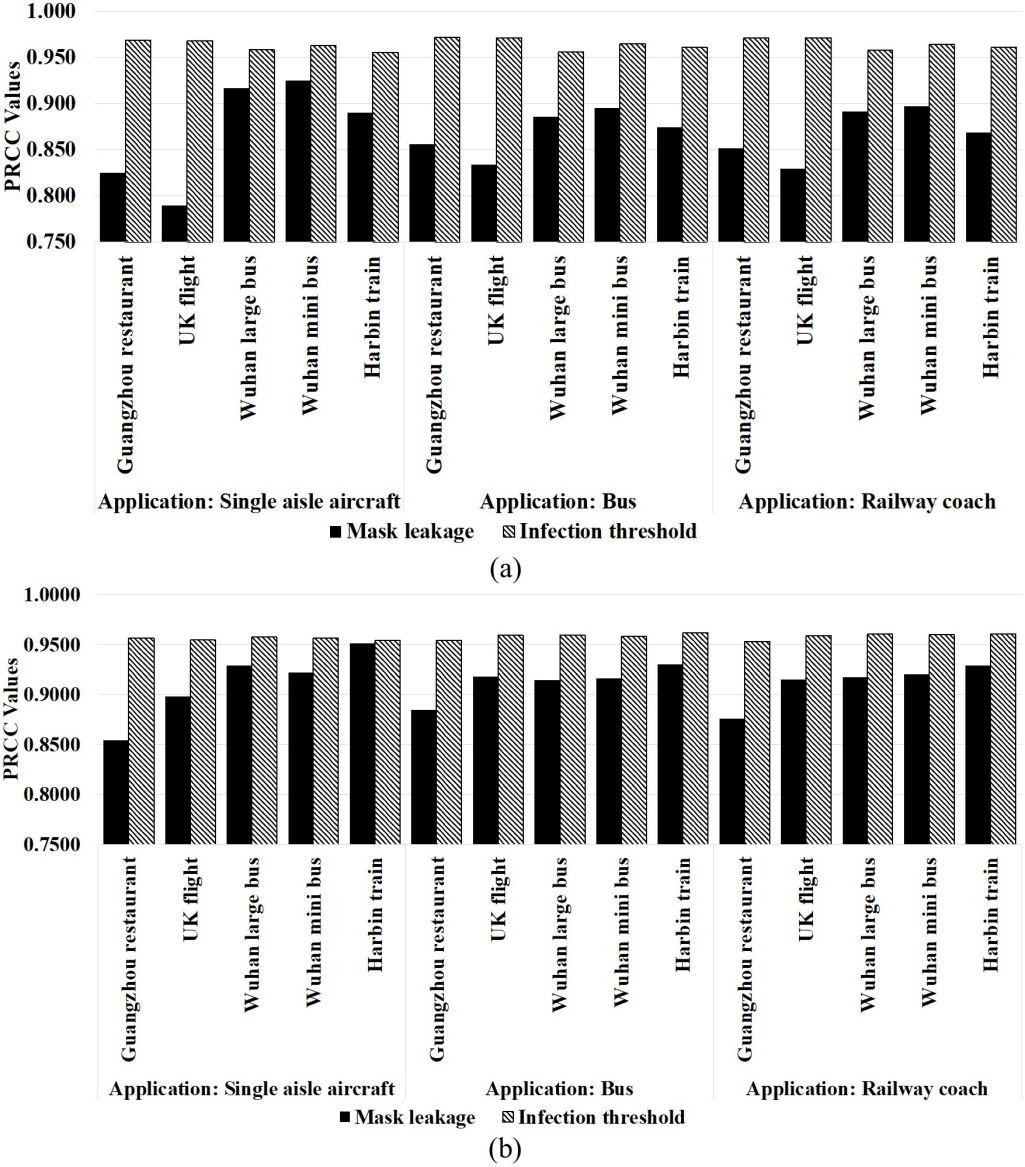
PRCC values of the number of infections in multiple layouts utilizing (a) the exponential models and (b) the Weibull models.

Sensitivity analysis can provide a quantitative perspective on how the infection outcomes of dose response models react to the variation of masking parameters. We used the sampling based Partial Rank Correlation Coefficients (PRCC) sensitivity analysis following the method described in Mubayi et al. [54] and Mubayi [55] to examine (i) which of the two mask related parameters, distance threshold and mask leakage, influence the outcomes more significantly, (ii) how the relative influence of the two parameters varies with dose levels and layout variations. The results shown in Fig 6 indicate that both input parameters have a positive PRCC that is significantly different from 0, with p-values in the range of 10^−20^ ~ 10^−40^ (p-value ≪ 0.05). This indicates that both parameters have a positive impact on the outcome i.e., there will be a higher infection risk with a larger infection threshold and more mask leakage. A higher value of PRCC indicates greater impact on the outcome. Between the two parameters, infection threshold, with |PRCC| > 0.9 at p < 0.05 is the more critical parameter in determining the magnitude of infections regardless of the dose levels and seating layouts. The infection threshold possesses a narrower PRCC distribution, whereas the PRCC of the mask leakage varies more widely depending on the dose response model, the dose amount, and the seating layouts. Mask leakage is less influential in high-dose scenarios using Guangzhou restaurant and the UK flight parameterization for all the layouts. Among the seating layouts, mask leakage is less influential in the more densely packed single aisle aircraft configuration. Beta- Poisson and Weibull models also exhibit similar trends.

Comparing between distance threshold and leakage, the higher PRCC for the distance threshold indicates that the effect of masks in reducing the threshold distance resulted in fewer infections than their effect in reducing the dose itself. PRCC for the mask leakage is lower in the high dose cases compared to intermediate and low dose situations. This suggests that the difference in mask quality is a more significant factor in low dose scenarios compared to high dose cases and larger more densely packed layouts.

## Discussion

Despite much literature focusing on SARS-COV-2 transmission, precise quantification of the amount of virions needed to trigger a successful infection is not known. Inherent biological stochasticity combined with differences in intervention usage leads to a wide variation in susceptibility of individuals. Variations in dose levels have resulted in varying outcomes of infection spreading with similar numbers of infective individuals in comparable situations [56]. Understanding and modeling this variability is crucial to effectively model infectious disease spread and for the design of mitigation measures.

Mathematical modeling of multivariate phenomena like infectious disease spread can explicitly account for biological, physical, or social mechanisms through mechanistic modeling. Alternately, phenomenological models extract information from real-world data to help capture the complex causal relationships without requiring the details of the underlying mechanisms. Phenomenological models are especially useful when details (e.g., dose threshold for infection) are not well defined. Here, we use a phenomenological modeling approach and fit different dose-response relations to empirical data of infectious disease spread in transportation. The dose levels in these transportation scenarios vary enabling parametrization of models at different dose levels.

We find that three dose response relations based on exponential, beta-Poisson and Weibull distributions can adequately reproduce the empirical data with appropriate parameterization. Weibull model differs from the other two due to the differences in the tails of the distributions in parameter sweeps and sensitivity analysis.

When modeling is done for prescriptive purposes e.g., for identify interventions, a large number of scenarios are needed, and the intervention needs to be adequate in all (or most) of these scenarios to be considered robust. In addition, it is useful to model extreme situations with high dose parameterization and show that the mitigation measures are effective in such conditions. By using different infection models parametrized to different levels of doses, one can bound the outcomes of hypothetical studies leading to a better understanding. For example, consider the mitigation measure of mask usage. We find that N95 masks are extremely effective in reducing infection spread in different transportation applications, using different parameterizations and models. Lower quality masks are also effective but for intermediate and low dose scenarios. This observation aligns well with meta-analysis of mask mitigating effect, such as Chu et al. [57] which considers and compares N95 masks and regular masks based on a review of 216 studies. Sensitivity analysis indicates that the effect of mask in reducing infection distance threshold is more crucial than in reducing the quantity of the dose itself. Additionally, we find that mask quality has a more significant impact in intermediate and low dose scenarios, compared to high dose case. Transportation systems often involve high-density crowds with diverse levels of intervention use. Our study indicates that high quality mask use is an effective mitigation measure in different transportation modes.

We compare the utilization of the exponential model and the Weibull model in analyzing the mitigating factors in high-dose and intermediate dose scenarios. The parameter sweep in Figs 4 and 5 suggests that, for both high-dose and intermediate-dose situations, the two models produce similar infection outcomes for the most unprotected scenario and protected (N95) scenarios in all three generic transportation contexts. However, there is a difference in the infection reduction estimated by the two models for some variations of mask usage parameters. For example, one can consider the scenario of poorly adhered N95 masks, i.e., 97% filtration and 3.5m spreading distance. For high-dose or intermediate dose situations, exponential model suggests about 50% reduction of infections, whereas the Weibull model suggests 80% reduction.

To determine which model is superior for more accurate predictive modeling, we need experimental or empirical data. For example, to determine if 50% reduction or 80% reduction is more accurate, we need empirical data on the infection patterns with mask usage variations. Such data is not often available. Despite this limitation, models can still be used for prescriptive purposes. For this purpose, one would generate a variety of possible scenarios and identify interventions that are effective under all these scenarios, so that the intervention is conservative and robust against the inherent uncertainties due to biological stochasticity and human behavior. In the above example, the Weibull model exhibits higher reduction in infections with improper mask use compared to the more conservative estimate obtained using exponential model. Therefore the exponential model is better suited for prescribing mitigation policy.

The limitations of the study are as follows: (a) We did not include dynamic evolution of contacts due to movement of people. (b) The infection spread events considered for model parameterization are early in the pandemic, therefore, we did not include data corresponding to different variants of concern, or the effect of vaccination. (c) Our contact analysis is limited to the transportation event, and does not consider the possibility that transmission might have occurred through prior interactions, e.g. at boarding gate. (d) In future work, we intend to enhance our models incorporating dynamic contact analysis based on pedestrian movement. Additionally, we aim to utilize computational fluid dynamics models, integrating details of virus shedding distributions specific to transportation contexts. This approach is intended to refine and augment the applicability of our models.

## Conclusion

We parameterized three common dose-response models with empirical events of SARS-COV-2 transmission to generate model parameters corresponding to high-, intermediate-, and low-dose scenarios. We then used parameterized models to analyze the spread of SARS_COV-2 and the effect of mask usage in generic transportation modes, including a single aisle airplane, bus, and railway coach. We found that dose level had a significant impact on the number of secondary infections. The exponential model is conservative compared to the Weibull model for similar parameterization; therefore, it is better suited as a prescriptive model. While any mask use reduces secondary infections, high-quality N-95 masks are especially effective. Lower-quality masks exhibit a limited mitigation efficiency, especially for high-dose conditions. The PRCC sensitivity analysis indicated that the effect of masks in reducing the infection distance threshold is a key factor.

## Supporting information

S1 Data The raw data used for parameterization events are provided in the supplementary data. (ZIP)
